# Supplementary material for: Dual role of a GTPase conformational switch for membrane fusion by mitofusin ubiquitylation
Source: Life Sci Alliance. 2019 Dec 19;3(1):e201900476. doi: 10.26508/lsa.201900476 (PMC6925385; doi:10.26508/lsa.201900476)
Supplement: Supplementary file 1 [file LSA-2019-00476_TableS1.doc]

Table S1. Lysine residues ubiquitylated in Fzo1.

| <!--Col Count:11-->Protein | Number of GlyGly (K) | Amino acid | PEP | Score | Modified sequence | GlyGly (K) probabilities | Position in peptide | Charge | Intensity | Ratio mod/base |
| --- | --- | --- | --- | --- | --- | --- | --- | --- | --- | --- |
| YBR179C | 1 | K | 0,000223 | 140,1 | _DLSPETYK(gl)R_ | DLSPETYK(1)R | 8 | 3 | 1,6E+09 | 0,25112 |
| YBR179C | 1 | K | 7,79E-143 | 335,3 | _SSNSK(gl)AHLISSQLSQWNYNNNR_ | SSNSK(1)AHLISSQLSQWNYNNNR | 5 | 3 | 9,9E+08 | 6,484 |
| YBR179C | 1 | K | 0,0231033 | 111,5 | _K(gl)ESNLLSIK_ | K(1)ESNLLSIK | 1 | 2 | 2,1E+08 | 0,040552 |
| YBR179C | 1 | K | 0,0426789 | 91,31 | _LLSDIIMISK(gl)SNMK_ | LLSDIIMISK(0.96)SNMK(0.04) | 10 | 3 | 1,5E+08 | 0,50756 |
| YBR179C | 1 | K | 0,001189 | 100,2 | _ALSK(gl)LFHSQIVSVTNHLNALK_ | ALSK(1)LFHSQIVSVTNHLNALK | 4 | 4 | 1,3E+08 | 1,3063 |
| YBR179C | 1 | K | 1,89E-13 | 138,8 | _EAIDMYSIQNPK(gl)TYEIHTLK_ | EAIDMYSIQNPK(1)TYEIHTLK | 12 | 3 | 7,1E+07 | 0,10725 |
| YBR179C | 1 | K | 6,48E-10 | 173,7 | _EK(gl)LHVLQLNIK_ | EK(1)LHVLQLNIK | 2 | 2 | 3,7E+07 | NaN |
| YBR179C | 1 | K | 0,0071871 | 76,14 | _EK(gl)NGFNIEK_ | EK(1)NGFNIEK | 2 | 2 | 0 | NaN |
| YBR179C | 1 | K | 6,39E-06 | 99,82 | _KFDK(gl)IRDK_ | KFDK(1)IRDK | 4 | 2 | 0 | NaN |
| YBR179C | 1 | K | 5,61E-107 | 153 | _K(gl)LQELDYIHLNAQR_ | K(1)LQELDYIHLNAQR | 1 | 2 | 0 | 0 |
